# *In vitro* silencing of the insulin receptor attenuates cellular accumulation of fibronectin in renal mesangial cells

**DOI:** 10.1186/1478-811X-10-29

**Published:** 2012-10-12

**Authors:** Naohiro Yano, Daisuke Suzuki, Masayuki Endoh, Weizhi Zhang, Yan Chun Xu, James F Padbury, Yi-Tang Tseng

**Affiliations:** 1 Department of Pediatrics, Women & Infants Hospital, The Warren Alpert Medical School of Brown University, Providence, RI, 02905, USA; 2 Division of Nephrology and Metabolism, Tokai University School of Medicine, Isehara, Kanagawa, 259-1193, Japan; 3 Department of Cardiothoracic Surgery, The Second Xiangya Hospital, Central South University, Changsha, China

**Keywords:** Insulin receptor, Fibronectin, Mesangial cells, CREB-1, MMP-9

## Abstract

**Background:**

Insulin receptor (InsR) and insulin signaling proteins are widely distributed throughout the kidney cortex. Insulin signaling can act in the kidney in multiple ways, some of which may be totally independent of its primary role of the maintenance of whole-body glucose homeostasis. However, descriptions of the insulin signaling in renal glomerular mesangial cells (MCs) are quite limited and the roles of insulin signaling in MC functions have not been sufficiently elucidated.

**Results:**

InsR silencing induced a unique phenotype of reduced fibronectin (FN) accumulation in renal glomerular MCs. Transcription level of FN was not significantly changed in the InsR silenced cells, suggesting the phenotype switching was caused by post-transcriptional modification. The decreased expression of InsR was associated with enhanced activity of insulin-like growth factor-1 receptor (IGF-1R)/PI3K/Akt signaling pathway which contributed in part to the attenuation of cellular FN accumulation. Formation of IGF-1R homodimer was increased in the InsR silenced cells. The InsR silenced cells also showed increased sensitivity to exogenous IGF-1, and increased PI3K activity was reversed significantly by incubating cells with IGF-1R specific antagonist, AG538. PI3K/Akt dependent activation of cAMP responsive element-binding protein (CREB)-1 induced expression of matrix metalloproteinase (MMP)-9 and suppressing MMP activity by doxycycline partially reversed FN accumulation in the InsR silenced cells.

**Conclusions:**

The effects of InsR silencing on cellular FN accumulation *in vitro* are, at least partially, mediated by increased degradation of FN by MMPs which is induced by enhanced signaling sequence of IGF-1R/PI3K/Akt/CREB-1.

## Background

One of the common local pathologic changes of glomerulonephropathy is accumulation of extracellular matrix (ECM) components, including fibronectin (FN), which results in glomerulosclerosis. Although the mechanisms responsible for the ECM protein deposition are still inconclusive, the role of renal glomerular mesangial cells (MCs) in this sclerotic change has been gathering increasing attentions. Glomerular mesangium is an area which shows the most prominent ECM accumulations in diseased kidney. And the resulting glomerular fibrosis has been recognized as the major degenerative event in glomerulonephropathies regardless of their etiologies [1-3]. MCs are specialized pericytes located among the mesangium area within the renal corpuscle of the kidney [4]. ECM protein deposition could be caused by matrix deposition exceeding matrix degradation. Many studies have focused on a possible imbalance of in situ synthesis and degradation of ECM proteins in glomeruli [5,6]. Indeed, some animal experiments have shown that the accumulation of ECM proteins in focal segmental glomerulosclerosis is associated with increased ECM protein synthesis coupled with suppression of expression or activity of proteolytic enzymes, including cathepsins and matrix metalloproteinases (MMPs) [6-8]. Mesangial cells secrete MMPs that degrade intact glomerular basement membrane, gelatin, soluble type IV collagen and FN at neutral pH [5,9]. MMPs and their specific inhibitors, tissue inhibitor of metalloproteinases (TIMPs), play an important role in regulating glomerular matrix remodeling [5,9,10]. Based on these findings, besides their three primary functions, that is, filtration, structural support, and phagocytosis, MCs have been postulated to be a key player for FN regulation in the kidney and is speculated as one of the major contributors to the sclerotic lesion in glomeruli [11].

The insulin receptor (InsR) and insulin signaling proteins are widely distributed throughout kidney cortex [12,13]. Insulin signaling can act in the kidney in multiple ways, some of which may be totally independent of its primary role of the maintenance of whole-body glucose homeostasis. As for renal tissue, the roles for insulin signaling in tubular epithelial cells have been described extensively in previous studies. The signaling is clearly anti-natriuretic, affecting sodium reabsorption in the proximal tubule [14], thick ascending limb [15,16], and collecting duct [17]. In contrast, descriptions of insulin signaling in MCs are limited and the roles of insulin signaling in MC functions have not been sufficiently elucidated.

The InsR and IGF-1R are structurally related transmembrane glycoproteins with approximately 50% amino acid sequence identity [18]. Post-translational processing results in dimerization and disulphide linkage of proreceptors. This is followed by proteolytic cleavage, which generates α and β subunits [19]. Mature and functional receptors thus have the subunit composition of (αβ)_2_. The extracellular α subunit contains a ligand binding site and the transmembrane β subunit possesses tyrosine kinase activity. The distinct physiological functions of insulin and IGFs depend on differences in the distribution and/or signaling potential of their respective receptors. Despite of this, it has been shown that a proportion of InsR and IGF-1R assemble as hybrid structures containing an (αβ) half of the InsR disulphide-linked to an (α’β’) half of the IGF-1R [20-22]. These hybrid receptors are functional, in that they bind IGF-1 with high affinity and insulin with somewhat lower affinity [23,24], and display IGF-1-induced autophosphorylation both *in vitro* and *in situ*[24,25]. MCs express both InsR and IGF-1R [11,26] and therefore have the potential to form functional hybrid receptors. However, the significance of hybrid receptors in MCs remains unclear.

Here, we explore the roles of InsR/IGF-1R signaling in cellular FN accumulation, using a SV40 immortalized mouse mesangial cell line, MES-13. With silencing InsR expression by shRNA, the cells showed significant reduction of cellular FN accumulation. The aim of the present work was to identify responsible alterations of the signaling pathway for the phenotype switching. We provide evidences that rearrangement in the balance between insulin and IGF-1 receptor homodimers and hybrid receptors contributed to the phenotype switching. We also demonstrate cAMP responsive element-binding protein (CREB-1) and MMP-9 as downstream factors for the attenuation of cellular FN accumulation.

## Results

### InsR silencing in MES-13 cells

**Figure 1:**
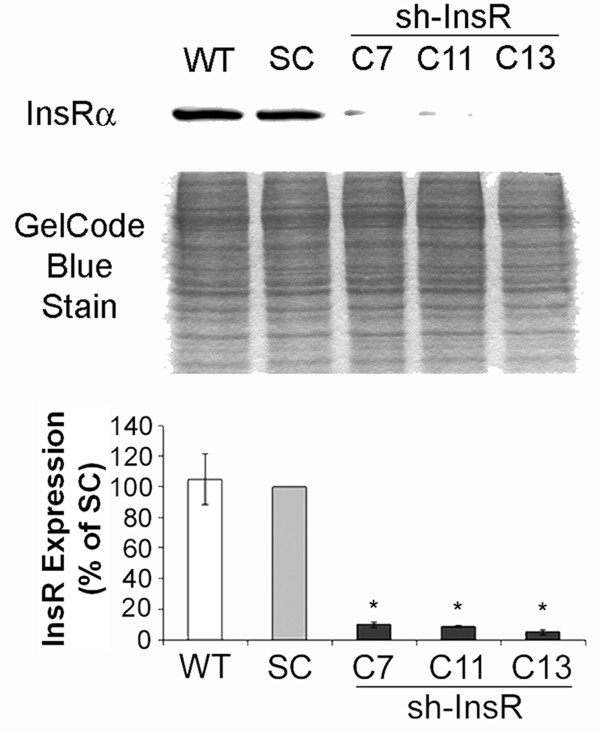
**Stable transfection of shRNA against InsR in MES-13 mesangial cells.** Cells (90% confluent) were stably transfected as described and serum-deprived for 16 hours. Immunoblotting with anti-InsRα antibody was performed on three clones of InsR silenced cells (clone 7, 11, 13) along with scrambled oligo (SC) transfected cells. An image of gel stained after transfer was shown as a loading monitor (middle panel). InsR expression levels were evaluated by densitometric analysis. Data are means ± SEM from four independent experiments. *, *P*<0.01 versus SC transfected cells, WT, wild type.

Twenty clones of shRNA against InsR transfected cells were tested for their phenotypes. Cells were seeded in 2 × 10^5^/ml density. The next day, serum was deprived from the medium for 16 hours. The quiescent cells were harvested and lysates were subject to Western blotting for InsRα. Three clones (#7, 11 and 13) which showed more than 85% suppression of InsR expressions were employed in the experiments (Figure 1).

### Cellular FN accumulation is attenuated in the InsR silenced cells

**Figure 2:**
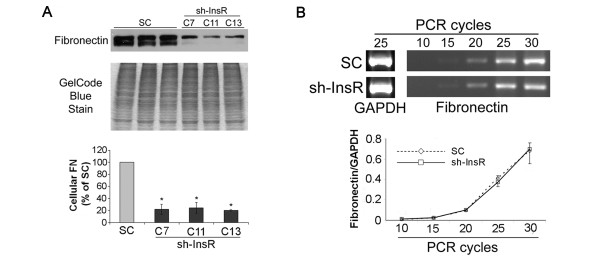
**Silencing of InsR attenuated cellular accumulation of FN in MES-13 mesangial cells.** (**A**) Immunoblotting with anti-FN antibody was performed on clones of InsR silenced cells and SC transfected cells. An image of gel stained after transfer was shown as a loading monitor (middle panel). Cellular FN levels were evaluated by densitometric analysis. Data are means ± SEM from four independent experiments. *, *P*<0.01 versus SC transfected cells (lower panel). (**B**) Semi-quantitative analysis of FN mRNA levels in InsR silenced and SC transfected MES-13 cells. Total RNA isolated from quiescent cells was subject to RT–PCR using FN and GAPDH (internal control) specific primers with the indicated number of amplification cycles. One representative image from four independent experiments is shown (upper panel). FN transcription levels were evaluated by densitometric analysis. Data are means ± SEM from four independent experiments.

Cell lysates from InsR silenced or scrambled oligo plasmid (SC) transfected cells were harvested as described above, and then served for Western blotting or semi-quantitative RT-PCR for FN. The InsR silenced cells showed remarkably decreased cellular FN accumulation, but no changes in transcriptional level of FN (Figure 2A, B). This finding suggested that the attenuation of cellular FN accumulation in InsR silenced cell is due to post-transcriptional modification.

### Activation of PI3K/Akt pathway and suppression of Ras/Erk1/2 in the InsR silenced cells

**Figure 3:**
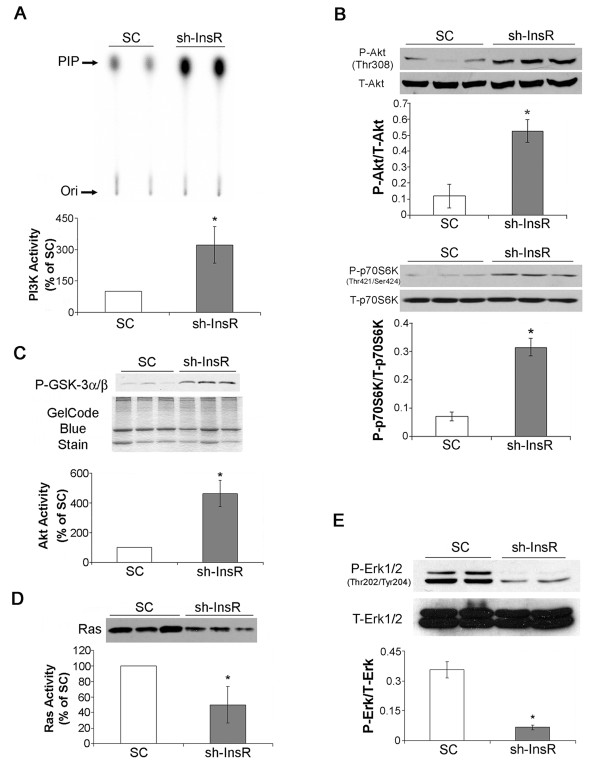
**Silencing of InsR enhanced PIK3/Akt signaling while suppressing Ras/Erk1/2 activities in MES-13 mesangial cells.** (**A**) Cell lysates were immunoprecipitated with an antibody againts phosphorylated-tyrosine, and PI3K activity was determined with *in vitro* lipid kinase assay. PIP, Phosphoinositide 3-phosphate, the phosphorylated end product. Ori, origin of migration in thin-layer chromatography (upper panel). The bar graph shows the densitometric scanning results (means ± SEM) from four independent experiments (lower panel). (**B**) Cell lysates were subject to Western blotting with antibodies against phosphorylated-Akt (P-Akt, Thr308), total-Akt (T-Akt), phosphorylated-p70S6K (P-p70S6K) and total-p70S6K (T-p70S6K). The bar graphs show the densitometric scanning results (means ± SEM) from four independent experiments. (**C**) Lysates were immunoprecipitated with an immobilized anti-phospho-Akt (Ser473) antibody, and the kinase reaction was carried out in the presence of cold ATP and GSK-3α/β fusion protein as described. An image of gel stained after transfer was shown as a loading monitor (middle panel). The bar graph shows the densitometric scanning results (means ± SEM) from four independent experiments. (**D**) Ras activation was evaluated by pulling down active GTP-loaded Ras with a GST fusion protein containing the Ras binding domain of Raf-1 (*GST-Raf1-RBD*) followed by blotting with an anti-Ras antibody (upper panel). The bar graph shows the densitometric scanning results (means ± SEM) from four independent experiments. (**E**) Cell lysates were subject to Western blotting with antibodies against phosphorylated-Erk1/2 (P-Erk1/2) and total Erk1/2 (T-Erk1/2). The bar graph shows the densitometric scanning results (means ± SEM) from four independent experiments. *, *P*<0.05 vs. SC transfected cells.

The cell lysates were subject to *in vitro* lipid kinase assay for measurement of PI3K activity. InsR silencing induced a significant increase in PI3K activity (Figure 3A). Phosphorylation of Akt and p70S6 kinase (p70S6K), two important downstream signaling factors, was also significantly increased indicating a general enhancement of PI3K/Akt signaling pathway in InsR silenced cells (Figure 3B). These results were confirmed by measurement of Akt activity (Figure 3C). Cell lysates were also subject to Ras pull-down assay and Western blotting for Erk1/2. InsR silencing induced significant decreases in Ras activity and phosphorylation of Erk1/2, indicating a suppression of Ras/Erk1/2 (Figure 3D,E).

### Attenuation of FN accumulation depends on PI3K/Akt signaling in the InsR silenced cells

**Figure 4:**
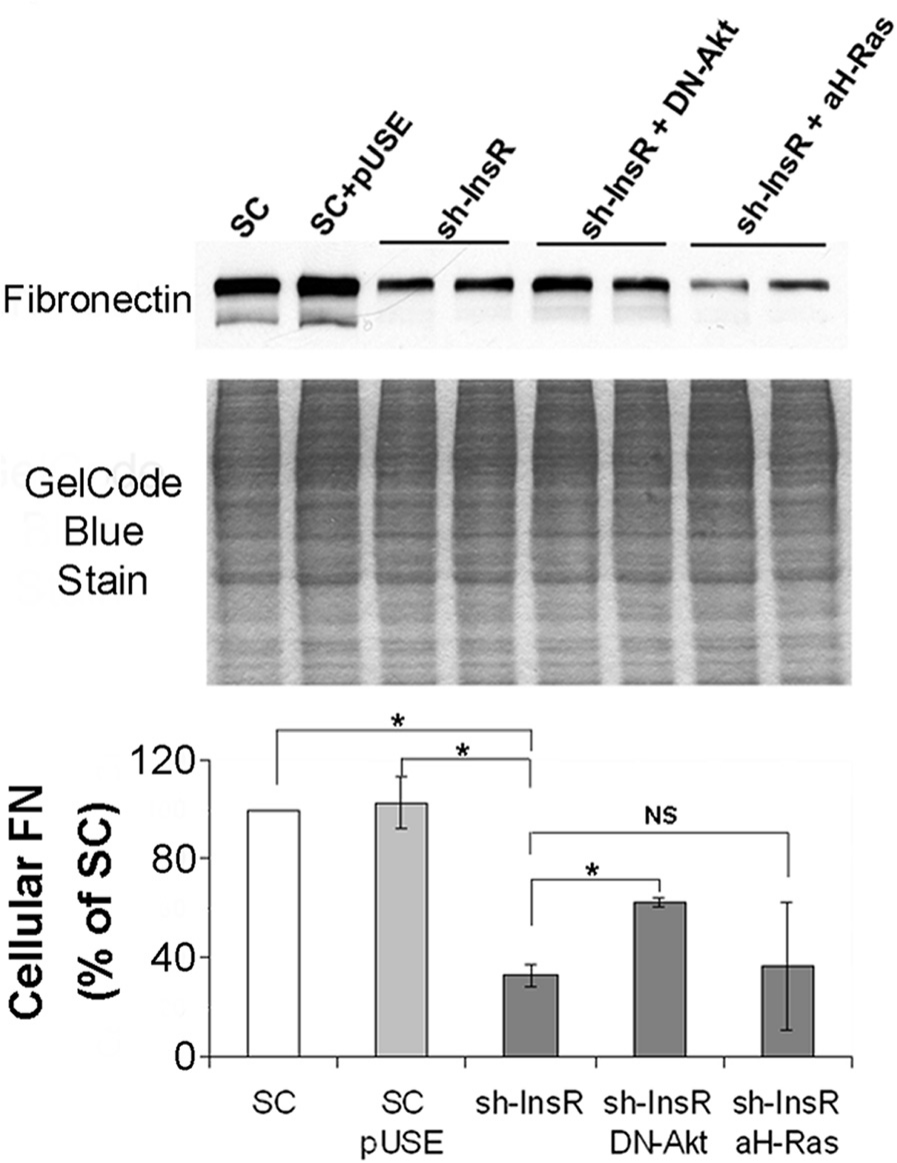
**InsR silencing induced reduction in cellular accumulation of FN was partially dependent on PI3K/Akt.** Cells were transfected with SC, sh-InsR, sh-InsR + dominant negative Akt (DN-Akt) cDNA or sh-InsR + activated H-Ras (aH-Ras) cDNA. Cell lysates were subject to Western blotting with an anti-FN antibody. A gel stained after transfer was shown as a loading monitor. The bar graph shows the densitometric scanning result (means ± SEM) from four independent experiments. *, *P*<0.01, NS, no significant difference.

In order to clarify how alteration in PI3K/Akt and Ras/Erk1/2 signaling pathways contributed to attenuation of cellular FN accumulation, we co-transfected a dominant negative (DN)-Akt or a constitutively activated H-Ras cDNA to the InsR silenced cells. Lysates from these cells were subject to Western blotting with anti-FN antibody to determine the effects of suppression of PI3K/Akt or enhancement of Ras/Erk1/2 on FN accumulation. As expected, silencing of InsR resulted in a significant reduction in the cellular levels of FN. The reduction, however, were partially reversed by co-transfection of a DN-Akt cDNA. In contrast, co-transfection of a constitutively activated-H-Ras cDNA did not influence InsR silencing induced reduction in FN levels (Figure 4). These finding suggest InsR silencing induced attenuation in FN is partially dependent on PI3K/Akt but not on Ras/Erk1/2 pathway.

### PI3K/Akt activation depends on IGF-1R signaling in the InsR silenced cells

**Figure 5:**
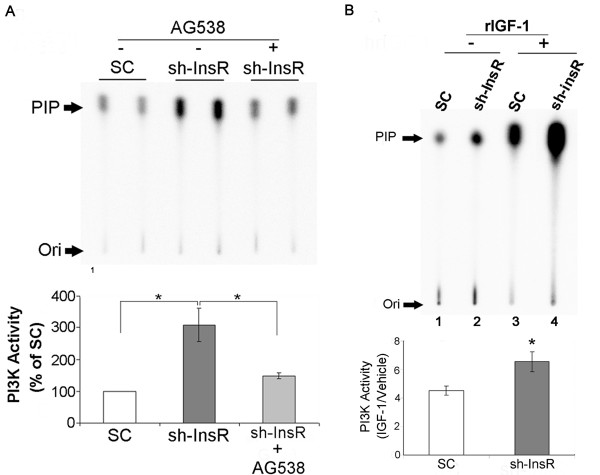
**PI3K activation in InsR silenced cells was dependent on IGF-1R signaling.** (**A**) Cells were incubated with AG538, an IGF-1R specific antagonist, or vehicle (DMSO) for 12 hours, and the cell lysates were subject to *in vitro* lipid kinase assay. PIP (phosphoinositide 3-phosphate), the phosphorylated end product. Ori, origin of migration in TLC. The bar graph shows the densitometric scanning results (means ± SEM) from four independent experiments. *, *P*<0.01 vs. SC transfected cells. (**B**) Cells were treated with vehicle (H_2_O) or recombinant IGF-1 (rIGF-1, 10ng/mL) for 5 minutes, and the cell lysates were subject to *in vitro* lipid kinase assay (upper panel). Cell sensitivity to extrinsic IGF-1 is expressed as the ratio of PI3K activity in IGF-1 treated cells to that of vehicle treated cells. Data are means ± SEM from four independent experiments. *, *P*<0.05 vs. SC transfected cells.

As mentioned above, MCs express both InsR and IGF-1R. In order to investigate the involvement of IGF-1R in the augmented PI3K/Akt signaling pathway in the InsR silenced cells, we performed the following two experiments. First, the cells were incubated with vehicle (DMSO) or an IGF-1R specific antagonist, AG538 (50μM) for 12 hours, and the cell lysates were subject to *in vitro* immunoprecipitation lipid kinase assay to measure PI3K activity. Pharmacological blockade of IGF-1R effectively reversed the effect of InsR silencing on reducing PI3K activity (Figure 5A). The finding suggests that the elevated PI3K activity in InsR silenced cells was mediated by IGF-1R.

Next, we investigated sensitivity of the cells to exogenous IGF-1. The cells were stimulated with vehicle (H_2_O) or a recombinant IGF-1 (rIGF-1, 10ng/mL) for 5mintes, and the cell lysates were subject to *in vitro* immunoprecipitation lipid kinase assay to measure PI3K activity. The ratio of PI3K activity in rIGF-1 treated cells and vehicle treated cells were used as an indicator for cell sensitivity to IGF-1. In SC transfected cell, PI3K activity was significantly increased by rIGF-1 pretreatment (lanes 1, 3, Figure 5B). In InsR silenced cells, however, the increase in PI3K activity in response to rIGF-1 was even more significant (lanes 2, 4, Figure 5B). These results suggest an increase in sensitivity to IGF-1 in InsR silenced cells.

### InsR silencing induces alteration in InsR/IGF-1R hybrid formation

**Figure 6:**
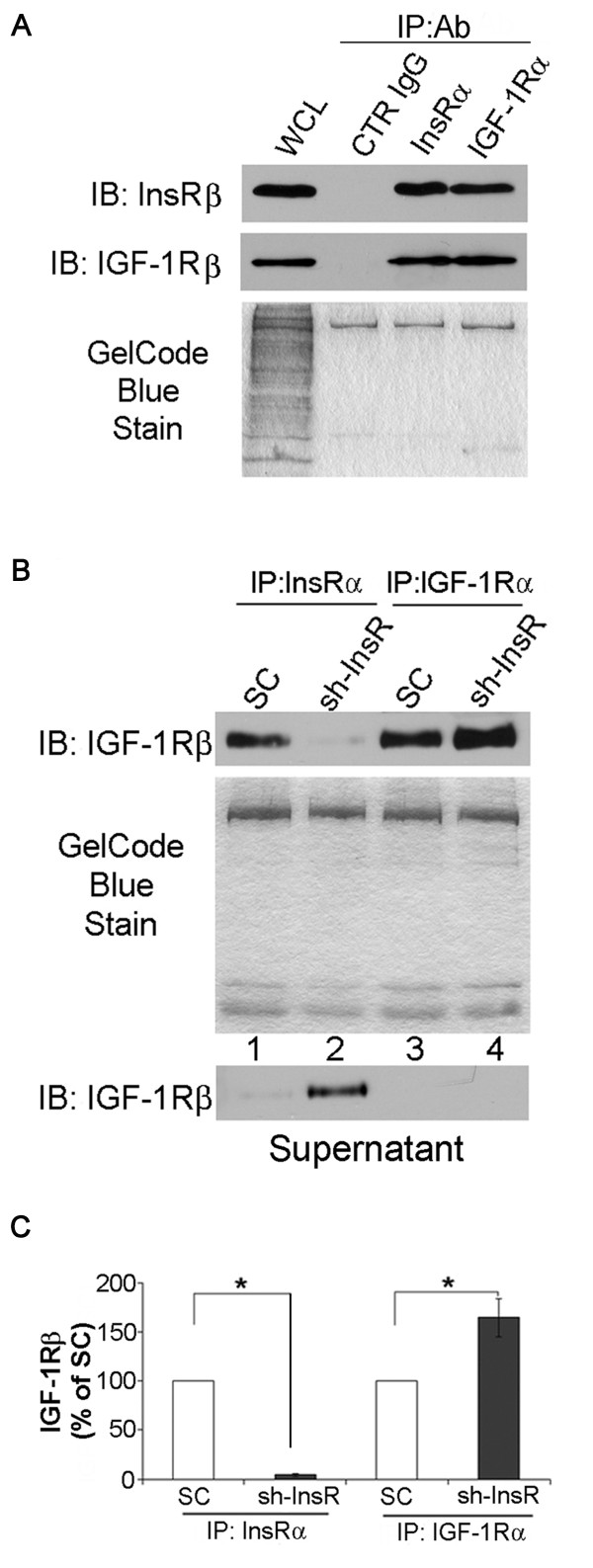
**Silencing of InsR disrupted InsR/IGF-1R hybrid receptor formation and increased IGF-1R homodimer formation.** (**A**) Lysates from non-transfected MES-13 mesangial cells were immunoprecipitaed (IP) using antibodies against IGF-1Rα, InsRα or control (CTR) IgG, an irrelevant control antibody. Immunoprecipitates were immuneblotted (IB) for IGF-1Rβ or InsRβ. WCL, whole cell lysate. An image of gel stained after transfer was shown as a loading monitor (lower panel). (**B**) Hybrid receptors in SC or InsR shRNA (sh-InsR) transfected cells were detected by immunoprecipitation with antibodies directed against either the InsRα or IGF-1Rα. Immunoprecipitated receptors were detected by immunoblotting with an anti-IGF-1Rβ antibody (upper panel). An image of gel stained after transfer was shown as a loading monitor. Immunodepletion was assessed by immunoblotting the supernatants from immunoprecipitate with an anti-IGF-1Rβ antibody (middle panel). The bar graph shows the densitometric scanning results (upper panel, means ± SEM) of four independents experiments *, *P*<0.05.

InsR and IGF-1R are known to be originated from a common ancestral gene and sharing structures which are similar enough to form a hybrid receptor. To determine whether or not formation of hybrid receptors play a role in the observed activation of IGF-1R/PI3K/Akt signaling pathway in the InsR silenced cells, two set of experiments were performed. First, co-immunoprecipitation experiments were carried out in wild type (non-transfected) MES-13 cells. The results verified that IGF-1R and InsR can be reciprocally co-immunoprecipitated (Figure 6A). This confirmed specific detection of hybrid receptors, which form via random dimerization as a function of the molar fractions of the IGF-1R and InsR [21,27,28]. Suppressed expression of InsR would therefore be expected to affect the balance between formation of hybrid receptors and homodimers. Next, lysates from the SC transfected cells and InsR silenced cells were immunoprecipitated with anti-InsRα or anti-IGF-1Rα antibody followed by immunoblotting with anti-IGF-1Rβ for co-immunoprecipitation of hybrid receptors. Suppression of InsR resulted in 1) decreased IGF-1R co-precipitation, consistent with reduction in hybrid receptor formation (lanes 1–2, upper panel, Figure 6B); 2) increased formation of IGF-1R homodimers (lane 3–4, upper panel, Figure 6B). The latter was confirmed by immunoblotting the supernatants with anti-IGF-1Rβ. Detection of IGF-1R was significantly increased in the anti-InsRα immunoprecipitated supernatant from InsR silenced cells, indicating increased formation of IGF-1R homodimers (middle panel, Figure 6B). Taken together, InsR silencing resulted in reduced formation in IGF-1R/InsR hybrid receptor but increased formation of IGF-1R homodimer in MES-13 cells. These alterations in the balance of InsR and IGF-1R homodimer and hybrid receptor might contribute to the observed changes in signaling pathways in the InsR silenced cells.

### CREB-1-mediated MMP-9 expression leads to increased degradation of cellular FN in the InsR silenced cells

**Figure 7:**
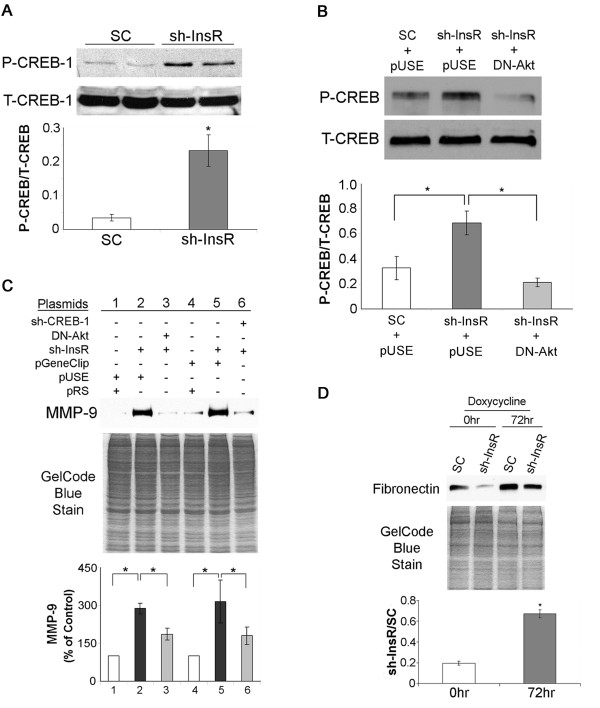
**Increased MMP-9 expression and CREB-1 phosphorylation in InsR silenced cells.** (**A**) Cell lysates from SC- and sh-InsR-transfected cells were subject to Western blotting using an antibody against phosphorylated (P-CREB-1) or total CREB-1 (T-CREB-1). The bar graph shows the densitometric scanning results (means ± SEM) from four independent experiments. *, *P*<0.01 vs. SC transfected cells. (**B**) Scrambled oligo (SC)- and sh-InsR-transfected cells were co-transfected with pUSEamp^(+)^ empty vector or a dominant negative (DN)-Akt vector. Cell lysates were subject to Western blotting for CREB-1 as described in (A). The bar graph shows the densitometric scanning results (means ± SEM) from four independent experiments. *, *P*<0.01(lower panel). (**C**) Cells were stably co-transfected with plasmids as indicated and lysates were subject for Western blotting for MMP-9. pRS, a control plasmid for InsR shRNA; pUSE, pUSEamp^(+)^, a control plasmid for DN-Akt; pGeneClip, a control plasmid for CREB-1 shRNA (upper panel). An image of gel stained after transfer was shown as a loading monitor (middle panel). The bar graph shows the densitometric scanning results (means ± SEM) from four independent experiments. *, *P*<0.01. (**D**) Scrambled oligo (SC)- and sh-InsR-transfected cells were incubated with doxycycline (10μg/mL, 72 hours) and cell lysates were subject to Western blotting for FN. An image of gel stained after transfer was shown as a loading monitor (middle panel). The bar graph shows the densitometric scanning results (sh-InsR/SC, means ± SEM) from four independents experiments. *, *P*<0.01.

In the process for exploring the downstream factors which are involved in altered FN accumulation, a couple of unique phenotypes in InsR silenced cells were indentified. First, phosphorylation of CREB-1 is enhanced in the InsR silenced cells (Figure 7A). The elevated phosphorylation was effectively reversed by shutting down Akt activity by transfecting a dominant negative (DN)-Akt cDNA (Figure 7B). Second, expression of MMP-9 was increased in InsR silenced cells, and the increased expression was partially dependent on Akt or CREB-1 activity (Figure 7C).

Next, we incubated cells with doxycycline (10μg/mL), a broad-spectrum MMP inhibitor, for 72 hours, and then evaluated intracellular FN by Western blotting. As expected cellular accumulation of FN in InsR silenced cells was about 20% of that of SC transfected cells. Inhibition of MMP with doxycycline in InsR silenced cells effectively reversed the reduction in intracellular FN levels to about 70% of that of SC cells (Figure 6D). Taken together, these findings suggest that the attenuated cellular accumulation of FN in the InsR silenced cells was partially dependent on increased degradation by MMP which was induced by IGF-1R/PI3K/Akt/CREB-1 signaling pathways.

## Discussion

In cells expressing both InsR and IGF-1R, InsR hemireceptors may heterodimerize with IGF-1R hemireceptors, leading to the formation of hybrid receptors (HRs). Heterodimerization of the two receptors is due to the high degree of homology between InsR and IGF-1R, which ranges from 27 to 84% depending on the region that is compared [27,29]. Heterodimerization is believed to occur with a similar efficiency as homodimerization. So the cellular proportions of homodimers and hybrids are supposed to be dependent on the expression ratios of each hemidimers [21-24,28,30]. Although, the physiological role of HRs is still unclear, early studies carried out with affinity chromatography-purified HRs indicated that these receptors mostly bind IGF-1 and that they bind insulin with much lower affinity [24,31].

Findings in the present study suggested that silencing InsR caused increased formation of IGF-1R homodimers in MCs. It’s possible that the change in the balance of the homodimers and hybrid receptors induced remarkable phenotypic changes of the attenuated cellular FN accumulation. Regardless of etiology, most end-stage glomerular diseases are characterized by accumulation of ECM proteins, including FN, in mesangium and other areas in glomeruli [1-3]. Over accumulation of the ECMs leads to sclerotic non-functional status of the kidney. So controlling the local ECM accumulation is believed to be one of the key therapeutic targets to prevent the development and progression of glomerular diseases. In spite of the extensive studies in the last decades, the mechanisms underlying this abnormal accumulation of ECM have not been fully elucidated. Our *in vitro* data suggested that alteration in the balance between InsR and IGF-1R and the resultant changes in PI3K/Akt signaling pathway contributed to the attenuation of cellular FN accumulation in MCs.

Expression of InsR is influenced by multiple factors. Estrogen showed inhibitory effect on *Ir* promoter activity [32]. In accordance with the negative effect of the estrogen, monocyte InsR content is higher during the luteal phases in adult females. This elevation is abolished by use of oral contraceptives or pregnancy [33]. Moreover, glucocorticoids and thyrotropin have been reported to enhance InsR expression while insulin down-regulates its cognate ligand [34-37]. Finally, nutrition and exercise have been reported to influence IR expression [38]. Various chronic glomerulopathy prone conditions such as poorly controlled diabetes, malignant tumors, systemic autoimmune diseases, pregnancy are inclinable to be accompanied endocrinological/metabolic disturbance which may affect expression of InsR or IGF-1R [39-42]. In these patients, appropriate control of the metabolic disturbance may potentially contribute to prevent the renal complications by correcting the InsR/IGF-1R balances.

IGF-1 has been shown to regulate protein synthesis in renal proximal tubular epithelial cells by activating both PI3K and Erk pathways [43]. Whereas, in the present study, InsR silencing caused increased PI3K activity and decreased Erk1/2 pathway activity. The reason for these discrepant findings has not been fully investigated but can be explained for the following: 1) we used renal glomerular MCs, which are specialized pericytes, whereas others used renal proximal tubular epithelial cells; 2) In our model, we showed silencing of InsR is associated with reduced Erk1/2 and increased PI3K activity. The latter can be blocked with IGF-1R inhibition. Hence, our experimental model is different from others that reported acute treatment with IGF-1 activates both PI3K and Erk1/2; 3) Altered InsR/IGF-1R balance may also be a factor for this characteristic phenotype of the InsR silenced MCs. Elucidating further details in the mechanisms will benefit understanding significance of InsR/IGF-1R balance in the fibrotie phenotype switching in MCs.

The ECM accumulation can result from either increased synthesis or decreased degradation of ECM components or both. MMPs are one of the main contributors to degrade ECMs. Quite a few numbers of mechanisms for regulation of MMP activities and expressions in various tissues have been reported and our study is the first to describe the involvement of InsR/IGF-1R signaling in MMP expression. We also showed CREB-1 as a specific transcription factor to regulate MMP expression in our model. Based on these findings, we proposed a novel signaling model for regulation of cellular FN accumulation. In this model, silencing InsR promotes increased formation of IGF-1R homodimer which alters the activation status of downstream kinases. Enhanced activity of PI3K/Akt induces activation of CREB-1 and results in increased MMP expression to promote FN degradation. Since the MES-13 cells synthesize IGF-1 ( Additional file 1) and since sensitivity to IGF-1 is increase in the InsR silenced MCs, in our *in vitro* model these endogenously produced IGF-1 possibly contributes to the observed changes in signaling pathways in an autocrine manner.

## Conclusions

In summary, we have identified the phenotype of reduced cellular accumulation of FN in InsR silenced MCs. We explored the mechanisms underlying this unique phenotype. Our data suggest that the altered balance in the formation of InsR/IGF-1R homodimer and hybrid receptor is a crucial factor in the phenotype switching in MCs. We also demonstrated that resultant change in PI3K/Akt signaling pathway was involved in the induction of this phenotype. We further established that PI3K/Akt induced CREB-1 activation lead to enhanced MMP-9 expression which was likely involved in reduced FN accumulation by enhancing degradation of FN. These findings provide important information for largely unknown mechanisms in InsR/IGF-1R mediated myofibroblast transdifferentiation (MFT) of MCs in glomerulopathies. Elucidating further details in this signaling pathway will benefit future choices of treatment for glomerulosclerosis.

## Materials and methods

### Cell culture

MES-13 mouse mesangial cells (ATCC #CRL-1927) were grown in a 3:1 mixture of Dulbecco's modified Eagle's and Ham's F-12 media (Invitrogen) supplemented with 5% (v/v) fetal bovine serum containing 50 units/ml penicillin G and 50 μg/ml streptomycin in a humidified atmosphere containing 5% CO_2_ at 37°C. Cells were first grown up to 90% confluence and synchronized overnight in serum-free medium prior to treatment. In some experiments cells were treated with recombinant IGF-1 (Invitrogen) or AG538 (EMD Chemical), an IGF-1R selective inhibitor, for the indicated durations.

### PI3K Assays

PI3K activity in cell lysate was determined with *in vitro* mmunoprecipitation lipid kinase assay as described previously [44]. Briefly, cell lysates (0.5 mg) were immunoprecipitated with anti-phosphotyrosine (anti-pY) antibody (Millipore), and L-α-phosphoinositide (Avanti Polar Lipids) was used as the lipid substrate (2 μg/reaction). After incubation, the final extracted reaction mixtures were spotted onto silica gel-coated TLC plates (Whatman) and run in TLC buffer (65% *n*-propanol, 0.54 M acetic acid).

### Antibodies

Antibody against fibronectin was purchased from Millipore. Antibodies against InsRα, InsRβ, IGF-1Rα and phospholylated or total CREB-1 were purchased from Santa Cruz Biotechnology. Antibodies against phosphorylated or total Akt, phosphorylated or total Erk1/2 and phosphorylated or total p70S6, IGF-1Rβ and MMP-9 were purchased from Cell Signaling Technology.

### Immunoprecipitation

For immunoprecipitation, protein lysates (0.5mg) were incubated with an antibody specific for InsRα or IGF-1Rα at 4°C for 4 hours with continuous rotation. After the incubation, a 20-μl packed volume of protein G-Sepharose (GE-Health Care) was added to the lysates and incubated for another 4 hours at 4°C. After washing, 45 μl of 1× Laemmli sample buffer was added to the beads. The sample was heated in boiling water for 5 minutes and quenched on ice for 2 minutes. After vortex and centrifuge, 20 μl of the supernatant was resolved on a 7.5% SDS-PAGE gel and immunoblotted with appropriate antibody.

### Pull down assay for Ras activity

Cells were lysed in Mg^2+^ IP buffer (25mM HEPES, pH7.7, 150mM NaCl, 1% Igepal CA-630, 10mM MgCl_2_, 1mM EDTA and 2% glycerol). Cleared cell lysates were normalized with the BCA assay (Santa Cruz Biotechnology). Normalized cell lysates were incubated with Raf-1 RBD preconjugated agarose (Millipore) for 2 hours at 4°C. Pelleted agarose were washed three times with IP buffer. Protein bound to the beads was eluted with 1xLaemmli’s buffer and subject to 12% SDS-polyacrylamide gel electrophoresis (SDS-PAGE). Ras was detected with a specific monoclonal antibody (clone Ras10, Millipore).

#### *In vitro* IP Akt activity assay

Cells were serum-starved for 16 hours, and then washed twice with phosphate-buffered saline and lysed in ice-cold lysis buffer (20mM Tris, pH 7.4, 150mM NaCl, 1% Triton X-100, 1mM EDTA, 1mM EGTA, 2.5mM sodium pyrophosphate, 1mM β-glycerolphosphate, 1mM sodium orthovanadate, 1μg/ml leupeptin, and 1mM phenylmethylsulfonyl fluoride). The extracts were centrifuged to remove cellular debris, and normalized by BCA assay (Santa Cruz). 250μg of protein from the lysate samples was incubated with gentle rocking at 4°C overnight with immobilized anti-phospho-Akt (Ser473) antibody cross-linked to sepharose beads (Cell Signaling Technology). After phosphorylated Akt was selectively immunoprecipitated from the cell lysates, the immunoprecipitated products were washed twice in lysis buffer and twice in kinase assay buffer (25mM Tris, pH 7.5, 10mM MgCl_2_, 50mM β-glycerolphosphate, 0.1mM sodium orthovanadate, and 2mM dithiothreitol), and the samples were resuspended in 40μl of kinase assay buffer containing 200μM ATP and 1μg of GSK-3α fusion protein. The kinase reaction was allowed to proceed at 30°C for 30 minutes and stopped by the addition of Laemmli SDS sample buffer. Reaction products were resolved by 15% SDS-PAGE followed by Western blotting with an anti-phospho-GSK-3α/β antibody (Cell Signaling Technology).

### Stable transfection

A vector expressing shRNA against InsR was purchased from Santa Cruz Biotechnology. A vector expressing shRNA against CREB1 was purchased from SABioscience. A vector expressing dominant negative Akt1 was engineered by inserting a K179M mutant dominant negative Akt1 cDNA (Millipore) into a multiple cloning site of a eukaryotic expression vector, pUSEamp(+) (Millipore). A 529F mutant constitutively activated H-Ras cDNA (Millipore) was processed similarly to engineer a vector expressing constitutively activated H-Ras. Stable transfection of the constructs in MES-13 cells was performed using Lipofectamine 2000 (Invitrogen) according to the manufacturer's instructions. Individual single cells were isolated and screened for neomycin or puromycin resistance. Phenotypes of the transfected cells were examined by Western blotting, IP Akt assay or Ras pull-down assay (Figure 1, Additional files 2, 3, 4).

### RT-PCR

Total RNA was isolated from cells using TRIzol reagent (Invitrogen). Reverse transcription was carried out on total RNA using SuperScript III (Invitrogen) followed by PCR using gene specific primers ( Additional file 5).

### Statistical analysis

Statistical significance of the difference among groups was analyzed by the paired Student’s t test or parametric ANOVA and Ryan’s multiple comparison test using Microsoft Exel (Microsoft) and ANOVA4 on the Web (http://www.hju.ac.jp/~kiriki/anova4/). All data were represented as the mean ± SEM of four different experiments unless otherwise noted in the figure legends. A probability of *p*<0.05 was considered to represent a significant difference.

## Competing interests

The authors declare that they have no competing interests.

## Authors’ contributions

NY designed and performed the experiments, analyzed the data, and wrote the paper. DS, ME, WZ, YCX, JFP and Y-TT critically revised the manuscript prior to the initial submission. All authors read and approved the final manuscript.

## Supplementary Material

Additional file 1 **MES-13 mesangial cells produce intrinsic IGF-1.** (A) Lysates from quiescent control (SC) and InsR silenced (sh-InsR) cells were subject to immunoblotting with antibody against IGF-1. An image of gel stained after transfer was shown as a loading monitor. (B) Total RNA isolated from quiescent cells was subject to RT–PCR using IGF-1 and GAPDH (internal control) specific primers. Click here for file

Additional file 2 **Phenotyping of activated H-Ras transfected cells.** (A) Ras activities of the InsR shRNA and activated H-Ras double transfected cells were evaluated by Ras pull-down assay as described under “Materials and methods”. Clones, #2, 4 and 8 were used for the experiments. (B) Phosphorylation of Erk1/2 of the selected clones were evaluated by Western blotting. P-Erk1/2, phosphorylated Erk1/2; T-Erk1/2, total Erk1/2. Click here for file

Additional file 3 **Phenotyping of DN-Akt transfected cells.** Akt activities of InsR sh-RNA and DN-Akt double transfected cells were evaluated by IP Akt activity assay as described under “Materials and methods”. Clones #3 and 7 were used for the experiments. Click here for file

Additional file 4 **Phenotyping of CREB-1 shRNA transfected cells.** CREB-1 levels in InsR and CREB-1 shRNA double transfected cells were evaluated by Western blotting. An image of gel stained after transfer was shown as a loading monitor. Clones #1, 4 and 5 were used for the experiments. Click here for file

Additional file 5 PCR primer sequences for fibronectin, IGF-1 and GAPDH. Click here for file
