# Supplementary figures and images for: In vitro silencing of the insulin receptor attenuates cellular accumulation of fibronectin in renal mesangial cells
Source: Cell Commun Signal. 2012 Oct 12;10:29. doi: 10.1186/1478-811X-10-29 (PMC3507851; doi:10.1186/1478-811X-10-29)

A

SC

sh-InsR

IGF-1

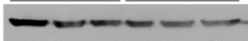GelCode  
Blue  
Stain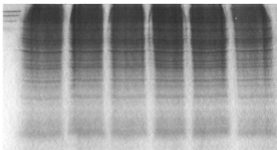

B

SC

sh-InsR

← GAPDH

← IGF-1

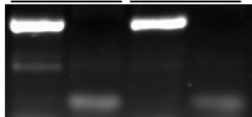

Supplement: Additional file 1 — MES-13 mesangial cells produce intrinsic IGF-1. (A) Lysates from quiescent control (SC) and InsR silenced (sh-InsR) cells were subject to immunoblotting with antibody against IGF-1. An image of gel stained after transfer was shown as a loading monitor. (B) Total RNA isolated from quiescent cells was subject to RT–PCR using IGF-1 and GAPDH (internal control) specific primers. [file 1478-811X-10-29-S1.pdf]

A

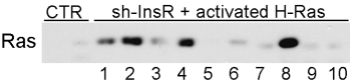

B

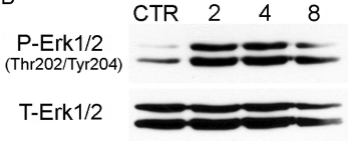

Supplement: Additional file 2 — Phenotyping of activated H-Ras transfected cells. (A) Ras activities of the InsR shRNA and activated H-Ras double transfected cells were evaluated by Ras pull-down assay as described under “Materials and methods”. Clones, #2, 4 and 8 were used for the experiments. (B) Phosphorylation of Erk1/2 of the selected clones were evaluated by Western blotting. P-Erk1/2, phosphorylated Erk1/2; T-Erk1/2, total Erk1/2. [file 1478-811X-10-29-S2.pdf]

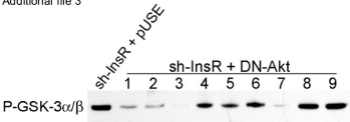

Supplement: Additional file 3 — Phenotyping of DN-Akt transfected cells. Akt activities of InsR sh-RNA and DN-Akt double transfected cells were evaluated by IP Akt activity assay as described under “Materials and methods”. Clones #3 and 7 were used for the experiments. [file 1478-811X-10-29-S3.pdf]

sh-InsR + sh-CREB-1

C 1 2 3 4 5 6 7

CREB-1

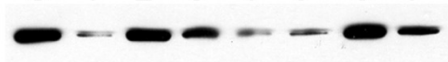

GelCode  
Blue  
Stain

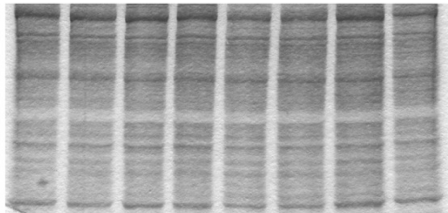

Supplement: Additional file 4 — Phenotyping of CREB-1 shRNA transfected cells. CREB-1 levels in InsR and CREB-1 shRNA double transfected cells were evaluated by Western blotting. An image of gel stained after transfer was shown as a loading monitor. Clones #1, 4 and 5 were used for the experiments. [file 1478-811X-10-29-S4.pdf]
