# Supplementary material for: In vitro silencing of the insulin receptor attenuates cellular accumulation of fibronectin in renal mesangial cells
Source: Cell Commun Signal. 2012 Oct 12;10:29. doi: 10.1186/1478-811X-10-29 (PMC3507851; doi:10.1186/1478-811X-10-29)
Supplement: Additional file 5 — PCR primer sequences for fibronectin, IGF-1 and GAPDH. [file 1478-811X-10-29-S5.pdf]

PCR primer sequences

Genes	Forward primers	Reverse Primers
Fibronectin	TGGAGGAGAACCAGGAGAG	GGTGTTGTAAGGTGGAATGG
IGF-1	ACAGGCTATGGCTCCAGCAT	GCTCCGGAAGCAACACTCAT
GAPDH	TGGCAAAGTGGAGATTGTTG	CTTCTGGGTGGCAGTGATG
